# Genetic profile and patient-reported outcomes in chronic obstructive pulmonary disease: A systematic review

**DOI:** 10.1371/journal.pone.0198920

**Published:** 2018-06-21

**Authors:** Hélder Melro, Jorge Gomes, Gabriela Moura, Alda Marques

**Affiliations:** 1 Lab3R – Respiratory Research and Rehabilitation Laboratory, School of Health Sciences, University of Aveiro, Aveiro, Portugal; 2 iBiMED – Institute for Biomedicine, School of Health Sciences, University of Aveiro, Aveiro, Portugal; 3 School of Engineering, Campus de Gualtar, University of Minho, Braga, Portugal; 4 Genome Sequencing and Analysis Lab, Department of Medical Sciences, University of Aveiro, Aveiro, Portugal; New York University School of Medicine, UNITED STATES

## Abstract

**Background:**

Chronic Obstructive Pulmonary Disease (COPD) impacts differently on patients at similar grades, suggesting that factors other than lung function may influence patients’ experience of the disease. Recent studies have found associations between genetic variations and patient-reported outcomes (PROs). Identifying these associations might be fundamental to predict the disease progression and develop tailored interventions. This systematic review aimed to identify the genetic variations associated with PROs in COPD.

**Methods and findings:**

Databases were searched until July 2017 (PROSPERO: CRD42016041639) and additional searches were conducted scanning the reference list of the articles. Two independent reviewers assessed the quality of studies using the Q-Genie checklist. This instrument is composed of 11 questions, each subdivided in 7 options from 1 poor-7 excellent. Thirteen studies reporting 5 PROs in association with genes were reviewed. Studies were rated between “good quality” (n = 8) and “moderate” (n = 5). The most reported PRO was frequency of exacerbations (n = 7/13), which was mainly associated with MBL2 gene variants. Other PRO’s were health-related quality of life (HRQOL) (n = 4/13), depressive symptoms (n = 1/13), exacerbation severity (n = 1/13) and breathlessness, cough and sputum (n = 1/13), which were commonly associated with other genetic variants.

**Conclusions:**

Although a limited number of PRO’s have been related to genetic variations, findings suggest that there is a significant association between specific gene variants and the number/severity of exacerbations, depressive symptoms and HRQOL. Further research is needed to confirm these findings and assess the genetic influence on other dimensions of patients’ lives, since it may enhance our understanding and management of COPD.

## Introduction

Chronic obstructive pulmonary disease (COPD) is a multifactorial, heterogeneous and progressive condition that affects 210 million people worldwide[1]. Severity of COPD is usually classified according to the degree of airway obstruction (assessed with spirometry), nevertheless it has been acknowledged that people at similar grades of COPD report different disease impacts[1]. These different reports among patients suggest that factors beyond lung function influence patients’ experience of the disease. Indeed, upstream factors, such as the presence of specific genetic variants, have already been reported to play a role in this matter[2]. For example, polymorphisms in *SERPINA1* usually lead to a deficiency of the α1 antitrypsin, affecting 1–2% of all COPD cases[3]. Additionally, the role of other candidate genes in the pathogenesis, comorbidities and outcomes of the disease have been studied[4].

Patient-reported outcomes (PROs) are a set of health outcomes directly reported by patients and may include symptoms (dyspnea, cough, pain, fatigue), exacerbation frequency and health status, among others [5]. These outcomes are accepted as the most faithful representation of patients’ perspectives of the impact of the disease and treatment benefits[6]. The needed of assessing PROs has been considerably highlighted in the Global Initiative for Chronic Obstructive Lung Disease (GOLD) 2017 update, which suggests that COPD classification should now be based on exacerbation frequency and patient’s perception of their symptoms rather than on lung function only [1].

In the last years, it has become more evident that there is a strong association between PROs and genetics, namely in lung cancer[7], which may suggest that strong associations may also exist between genetics and PROs in COPD. However, a review of the known associations has never been conducted. The combination of genetics and PROs would be valuable to identify patients susceptible to PROs deficits, understand the diagnosis, predict disease progression and develop tailored and timely interventions [8].

Therefore, the focus of this systematic review was to synthetize the genetic variations associated with different PROs in COPD.

## Methods

### Search strategy

The systematic review protocol was registered at Prospective Register of Systematic Reviews (PROSPERO) (ref CRD42016041639). A comprehensive systematic search was conducted in May 2016 and weekly updates were performed until July 2017 in the following medical databases: PubMed (1950–2016), Scopus (1960–2016) and Web of Science (1900–2016). The PICOS (Populations, Intervention, Comparison, Outcome and Study Design) framework was used to develop literature search strategies, however Intervention (I) and Comparison (C) terms were omitted as they were not applicable to the present review [9]. Accordingly, the search terms were based on a combination of the following keywords: [(COPD OR "chronic obstructive pulmonary disease" OR emphysema OR "chronic bronchitis") AND ("genetic associations" OR "genetic profile" OR "genetic analysis" OR gene) AND (dyspnea OR dyspnoea OR breathlessness OR fatigue OR cough OR depression OR anxiety OR "daily living" OR "quality of life" OR mood OR "well-being" OR “frequency of exacerbation” OR exacerbations OR "hospital admissions" OR "hospital length of stay" OR "acute exacerbations" OR “physical activity” OR "physical fitness" OR “physical function” OR “sputum production” OR phlegm OR pain OR “patient-reported outcomes” OR “patient-centered outcomes” OR “patient-centered outcomes”)]. The full search strategy can be found in supplementary material (S1 Table). The reference lists of the selected articles were also scanned for other potential eligible studies.

### Eligibility criteria

Studies were considered eligible if included adult patients (>18 years old) diagnosed with COPD and associated a genetic profile to one or more PRO. For the purpose of this systematic review, PRO were defined, according to the Cochrane Collaboration definition, as “reports coming directly from patients about how they feel or function in relation to a health condition and its therapy without interpretation by healthcare professionals or anyone else” [10]. Studies were excluded if they were conducted in animals, were written in languages other than English, Spanish, French or Portuguese and did not differentiate chronic obstructive diseases (i.e., presented pooled data from several chronic obstructive diseases such as asthma, COPD and bronchiectasis). Book chapters, review papers, abstracts of communications on meetings, letters to the editor, commentaries to articles, unpublished work and study protocols were not considered suitable and, therefore, were also excluded from this study. This systematic review was reported according to the Preferred Reporting Items for Systematic Reviews and Meta-Analysis (PRISMA) (S2 Table) [9, 11].

### Quality assessment

Quality, internal validity and risk of bias of the included studies were assessed using the Quality of genetic association studies checklist (Q-Genie) [12]. This instrument is composed of 11 questions to assess “rationale for study”, “selection and definition of outcome of interest”, “selection and comparability of comparison groups”, “technical classification of the exposure”, “non-technical classification of the exposure”, “other source of bias”, “sample size and power”, “a priori planning of analysis”, “statistical methods and control for confounding”, “testing of assumptions and inferences for genetic analysis” and “appropriateness of inferences drawn from results”. The Q-Genie checklist has 7 possible answers for each question (i.e., “1 (poor)”, “2”, “3 (good)”, “4”, “5 (very good)”, “6”, “7 (excellent)”). The overall quality of studies is classified as “poor quality” if score is ≤35, “moderate quality” if score is >35 and ≤45, and “good quality” (>45), for studies having control groups. For studies without control groups, values for the parameters listed above are ≤32, >32 and ≤40, and >40, respectively[12]. Two reviewers assessed the quality of studies independently. Disagreements were solved consulting a third reviewer.

### Studies selection and data extraction

First, duplicates were removed and one reviewer performed the initial screening of title, abstract and keywords of studies based on the type of publication and relevance for the scope of the review. Then the full-text of each potentially relevant study was screened for content to decide its inclusion in the review. For each accepted study, one reviewer extracted the following data to a previously structured table: last author’s name and year of publication, study design, sample characteristics (i.e., sample size, age, gender and COPD severity), PRO evaluated and outcome measures used, gene associated with the identified PRO and type of association between the PRO and the identified gene. Two independent reviewers further checked the extracted data for accuracy and completeness. Reviewers resolved disagreements by consensus.

### Data analysis

Consistency of the studies quality assessment (performed by the two reviewers), was explored with the Cohen’s kappa. The value of Cohen’s kappa vary from 0 to 1 and can be interpreted as: i) 0.00–0.20: slight agreement; ii) 0.21–0.40: fair agreement; iii) 0.41–0.60: moderate agreement; iv) 0.61–0.80: substantial agreement; v) 0.81–1.00: almost perfect agreement[13]. Statistical analysis was performed using IBM SPSS Statistics version 24.0 (IBM Corporation, Armonk, NY, USA).

## Results

### Search strategy

**Fig 1:**
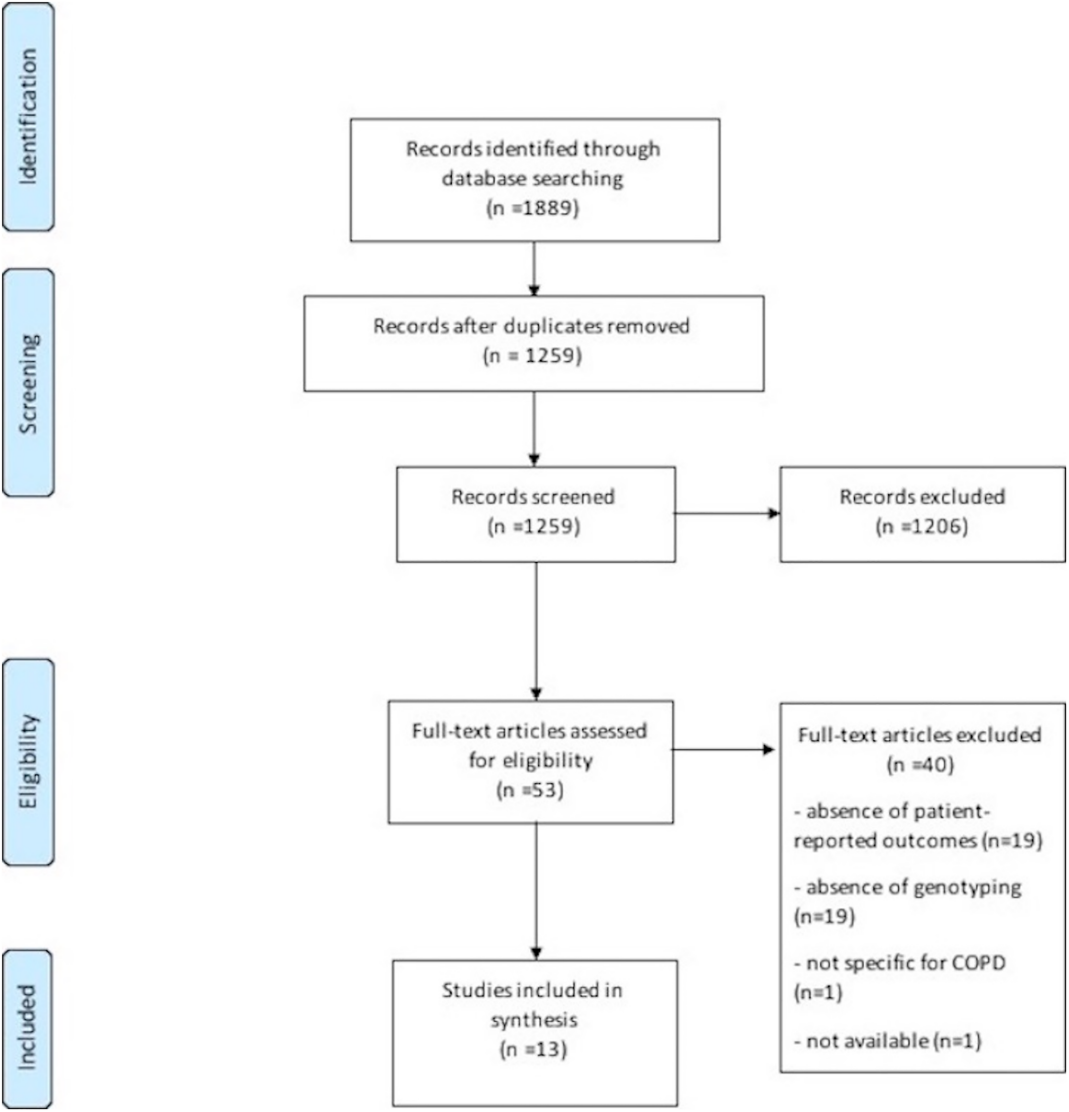
PRISMA flow diagram.

Database search identified 1889 studies of potential interest. After duplicates removal, 1259 articles were analyzed for relevant content. From these, 1206 were excluded due to the following reasons: non-original articles (n = 575), absence of PRO/genetic associations (n = 387), non-specific for COPD (n = 122), studies conducted in animals (n = 98), studies written in other languages rather than English, Spanish, French or Portuguese (n = 24). The full-text of the remaining 53 potentially relevant articles was assessed and 40 articles were excluded. Reasons for exclusion included: absence of PRO (n = 19), absence of genetic association (n = 19), not specific for COPD (n = 1) and unavailability of the article even after contacting the authors (n = 1). In total, 13 articles were included, all published in English. A detailed diagram of the review process is presented in Fig 1.

### Quality assessment

Articles scored between 39 and 55 in the Q-Genie checklist [12] (Table 1). Six articles were classified as studies without control group [14–19], from which five presented “good quality” and one was of “moderate quality”. The remaining articles, were classified as “studies with control group”, from which four were “moderate quality” and the remaining three of “good quality”. Items with the lowest classification were the “selection and comparability of comparison groups” and “sample size and power”. The agreement between the two independent reviewers was almost perfect (k = 0.83; 95% CI 0.29–1; p = 0.002).

**Table 1:** Quality assessment scores for the selected studies based on the quality of genetic association studies (Q-Genie).

Studies	Items											
	1	2	3	4	5	6	7	8	9	10	11	Score
**Bleecker et al,2012** [16]	5	5	Na	5	4	5	6	5	6	5	5	51
**Ishii et al, 2014** [25]	5	4	3	6	3	3	3	5	4	4	5	45
**Ishii et al, 2011** [21]	6	4	5	4	2	5	2	3	4	4	5	44
**Lin et al, 2011** [22]	5	6	3	5	2	3	3	5	2	4	4	42
**Mandal et al, 2015** [14]	6	6	Na	3	2	6	1	4	3	5	6	42
**Pillai et al, 2010** [15]	4	6	Na	5	5	4	5	6	5	4	6	50
**Quint et al,2011** [23]	5	4	5	5	2	3	6	3	5	4	5	47
**Quint et al, 2012** [24]	4	5	3	4	5	4	2	6	3	4	4	44
**Takabatake et al, 2006** [19]	7	6	Na	6	5	5	3	6	6	5	6	55
**Umeda et al, 2008** [17]	5	4	Na	4	4	5	2	4	4	3	4	39
**Yang et al, 2003** [20]	6	6	4	5	5	5	3	5	5	5	6	55
**Zhang et al,2015**[26]	5	5	5	4	4	5	4	6	4	4	4	50
**Zhang et al,2015** [18]	5	5	Na	4	4	4	5	6	4	4	5	46

1- rationale for study; 2- selection and definition of outcome of interest; 3- selection and comparability of comparison group (if applicable); 4- technical classification of the exposure; 5- non-technical classification of the exposure; 6 other sources of bias; 7- sample size and power; 8- a priori planning of analysis; 9- statistical methods and control for confounding; 10- testing of assumptions and inferences for genetic analysis; 11- appropriateness of inferences drawn from results. All items have a maximum score of 7.

Na–not applicable

### Study characteristics

Study characteristics are presented in Table 2. A total of 6520 patients with COPD with a mean age range of 63.2–71.8 years old, mainly males (4638 males– 71,13%) participated in the 13 studies included. Studies designs were observational (n = 9) [14, 15, 19–25] and pre and post intervention (n = 4)[16–18, 26].

**Table 2:** Patient reported outcomes, patient reported outcome measures and genetic associations assessed.

First author’s name & Year	Design	Participants	Population/Ethnicity	Patient Reported Outcome (PRO)	Patient Reported Outcome Measure (PROM)	Genes of interest	Single Nucleotide Polymorphisms	Results
Bleecker et al, (2012) [16]	Intervention, pre/post study	Study I: n = 1483 Gly/Gly: n = 575 (370 Male, 64%, 63±9 yrs) Smoking status: Ex/current: 319/256 Pack-years: n.a. FEV_1_ (%pred): 34.5±9.4 COPD grades (I/II/III/IV): n.a. Arg/Gly: n = 685 (433 Male, 63%, 63±9 yrs) Smoking status: Ex/current: 396/289 Pack-years: n.a. FEV_1_ (%pred): 34.4±9.2 COPD grades (I/II/III/IV): n.a. Arg/Arg: n = 223 (139 Male, 62%, 63±10 yrs) Smoking status: Ex/current: 130/93 Pack-years: n.a. FEV_1_ (%pred): 34.4±9.3 COPD grades (I/II/III/IV): n.a. Study II: n = 1383 Gly/Gly: n = 533 (373 Male, 70%, 64±9 yrs) Smoking status: Ex/current: 309/224 Pack-years: n.a. FEV_1_ (%pred): 33.9±9.1 COPD grades (I/II/III/IV): n.a. Arg/Gly: n = 635 (451 Male, 71%, 63±9 yrs) Smoking status: Ex/current: 373/262 Pack-years: n.a. FEV_1_ (%pred): 34.6±9.7 COPD grades (I/II/III/IV): n.a. Arg/Arg: n = 215 (133 Male, 62%, 63±9 yrs) Smoking status: Ex/current: 117/98 Pack-years: n.a. FEV_1_ (%pred): 34.1±9.6 COPD grades (I/II/III/IV): n.a	American; European; South African	HRQOL; Symptoms	SGRQ BCSS	ADRB2	rs1042713	BCSS: Study I (p≥0.378); Study II (p≥0.133) SGRQ: Study I (p = 0.909); Study II (p = 0.648)
Ishii et al, (2014) [25]	Observational study	n = 135 (127 Male, 94%, 69.3±7.9 yrs) Smoking status: Ex/current: 113/22 Pack-years: 74.5 ±47.6 FEV_1_ (%pred): 57.8±20.3 COPD grades (I/II/III/IV): 22/61/41/11	Japanese	Exacerbation frequency	Diary	GC	rs4588 rs7041	Exacerbation Frequency: **rs4588 SNP: p = 0.0048;** rs7041 SNP: p = 0.56.
Ishii et al, (2011) [21]	Observational study	n = 247: COPD (228 Male, 92%, 69.7±8.1 yrs) Smoking status; Ex/Current: 207/40 Pack-years: 69.4±42.6 FEV_1_ (%pred): 58.3±19.5 COPD grades(I/II/III/IV): n.a	Japanese	Depressive symptoms	HADS	SLC6A4	rs3794808 rs140701 rs140700 rs2020939 rs2020936	**rs3794808, p(adjusted)/ Depression score HADS: Trend: 0.016; Genotype: 0.052** rs140701, p(adjusted)/ Depression score HADS: Trend: 0.093; Genotype: 0.246 rs140700, p(adjusted)/ Depression score HADS: Trend: 0.559; Genotype: 0.844 rs2020939, p(adjusted)/ Depression score HADS: Trend: 0.13; Genotype: 0.261 rs2020936, p(adjusted)/ Depression score HADS: Trend: 0.903; Genotype: 0.966
Lin et al, (2011) [22]	Observational study	n = 84: Non-MBL -deficient genotypes (27 Male, 21%, 66.5±10.9 yrs) Smoking status: Ex/current: 60/24 Pack-years: 46 FEV_1_ (%pred): 46 COPD grades(I/II/III/IV): 2/28/39/17 n = 12: MBL-deficient genotypes (6 Male,50%,68.9±10.3 yrs) Smoking status: Ex/current: 10/2 Pack-years: 50 FEV_1_ (%pred): 41 COPD grades(I/II/III/IV): 1/4/6/1	Han Chinese (Taiwan)	Exacerbation frequency	Patient interviews	MBL2	rs11003125 rs7096206 rs1800451 rs5030737 rs1800450	**Frequency of infective exacerbation (times) in:** **Non-MBL-Deficient genotypes (n = 84): 3.52±0.78 Total episodes: 296** **MBL-Deficient Genotypes (n = 12): 4.75±1.22** **Total episodes: 57** **p˂0.0001**
Mandal et al, (2015) [14]	Observational study	n = 277 (190 Male, 84%, 67.8 ± 9.5 yrs) Smoking status: Ex/current: 203/74 Pack-years: Median 50 IQR (35–80) FEV_1_ (%pred): 48.2±17.5 COPD grades(I/II/III/IV): 0/129/104/40	European	Exacerbation frequency	Contacting the patients	MBL2	HL (-550 G>C; rs11003125) YX (-221 G>C; rs7096206) PQ (+4 C>T; rs7095891) A/D (+223 C>T; rs5030737) A/B (+230 G>A; rs1800450) A/C (+239 G>A; rs1800451)	MBL2 haplotype: HYPA Infrequent exacerbation (n = 87): Frequency: 0.282; Frequent exacerbation (n = 85): Frequency: 0.346; p = 0.13 MBL2 haplotype: LYQA Infrequent exacerbation (n = 75): Frequency: 0.243; Frequent exacerbation (n = 53): Frequency: 0.215; p = 0.49 MBL2 haplotype: LYPA Infrequent exacerbation (n = 30): Frequency: 0.097; Frequent exacerbation (n = 15): Frequency: 0.061; p = 0.16 MBL2 haplotype: LXPA Infrequent exacerbation (n = 71): Frequency: 0.230 Frequent exacerbation (n = 50): Frequency: 0.303; p = 0.50 MBL2 haplotype: LYPB Infrequent exacerbation (n = 27): Frequency: 0.087; Frequent exacerbation (n = 19): Frequency: 0.077; p = 0.77 **MBL2 haplotype: HYPD** **Infrequent exacerbation (n = 10): Frequency: 0.032 Frequent exacerbation (n = 21): Frequency: 0.085; p = 0.01** MBL2 haplotype: LYQC Infrequent exacerbation (n = 8): Frequency: 0.026; Frequent exacerbation (n = 3): Frequency: 0.012; p = 0.39
Pillai et al, (2010) [15]	Observational study	n = 1,609 (1086 Male, 67.5%, 63.8± 7.1 yrs) Smoking Status: Ex/Current, %: 64.5/35.5 Pack-years: 50.9±28 FEV_1_: 48.1±15.6 COPD grades(I/II/III/IV): n. a	European; Canadian; New Zealanders; Norwegians; Ukrainians; American	Exacerbation Frequency	Retrospective exacerbations: questionnaires Prospective exacerbations: telephone calls.	HHIP, FAM13A CHRNA3/5	rs13118928 rs8034191 rs7671167	Prior Exacerbations: rs13118928(HHIP): IRR: 0.877; 95% IC: 0.78–0.975; p = 0.015 rs8034191(CHRNA): IRR: 0.971; 95% IC: 0.869–1.084; p = 0.598 rs7671167(FAM13A): IRR: 1.081; 95% IC: 0.978–1.195; p = 0.129 **Prospective exacerbations** **rs13118928(HHIP): IRR: 0.906; 95 IC: 0.832–0.987; p = 0.024** rs8034191(CHRNA): IRR: 1.017; 95% IC: 0.930–1.113; p = 0.709 rs7671167(FAM13A): IRR: 1.028; 95% IC: 0.943–1.22; p = 0.528.
Quint et al, (2011) [23]	Observational study	n = 204 (119 Male, 58%, 70.7 ±11.1 yrs) Smoking status: Ex/current: 152/52 Pack-years: 51.6±38.7 FEV_1_ (l): 48.2±19.9 COPD grades(I/II/III/IV): 14/83/73/34	European	Exacerbation frequency	Diary	SERPINA1	11478G>A polymorphism	Exacerbation α_1_-antitrypsin: (GG): Median = 2.01 IQR (1.54–2.99); (GA/AA): Median = 1.98 IQR (1.67–2.12); GG vs. GA/AA: p = 0.75 baseline vs. exacerbation: (GG): p = 0.87; (GA/AA): p = 0.92
Quint et al, (2012) [24]	Observational study	n = 97 (61 Male, 62.9%, 71.8±8.8 yrs) Smoking status: Ex/current: 72/25 Pack-years: 50.7±34.2 FEV_1_:(%pred): 50.3±19.7 COPD grades(I/II/III/IV): n.a	European British)	Exacerbation frequency	Diary	VDR	rs1544410 rs731236 rs2228570	rs1544410(Bsml): Frequent exacerbators (n = 28): 3 (10.7%)–BB genotype; 12 (42.9%)–Bb genotype; 13 (46.4%)–Bb genotype. Infrequent exacerbators (n = 68): 15 (22.1%)–BB genotype; 26 (38.2%)–Bb genotype; 27 (39.7%)–Bb genotype p = 0.43 rs731236(Taql): Frequent exacerbators (n = 28): 10 (38.5%)–TT genotype; 13 (50%)–Tt genotype; 3 (11.5%)–Tt genotype. Infrequent exacerbators (n = 68): 24 (36.4%)–TT genotype; 29 (43.9%)–Tt genotype; 13 (19.7%)–Tt genotype p = 0.64 rs2228570(Fokl): Frequent exacerbators (n = 28): 10 (35.7%)–FF genotype; 14 (50,0%)–Ff genotype; 4 (14.3%)–Ff genotype. Infrequent exacerbators (n = 68): 21 (30.9%)–FF genotype; 38 (55.9%)–Ff genotype; 9 (13.2%)–Ff genotype p = 0.87
Takabatake et al, (2006) [19]	Observational study	n = 276 (276 Male, 100%, 72.9 ± 1.6 yrs) Smoking status: Ex/current: 276/0 Pack-years: n.a. FEV_1_: (%pred) n.a. COPD grades(I+II/III+IV): n.a.	Japanese	Exacerbation severity	Questionnaire	CCL1	rs2282691	Kaplan-Meier Genotype: AA; No. of patients:60 No. of events: 9 Survival Time, mo (95% CI): 28 (27–30) Log-Rankk Statistic: AA vs AT: 1.51 (0.2189) **Genotype: AT; No. of patients: 132** **No. of events:12** **Survival Time, mo (95% CI): 29 (28–30)** **Log-Rankk Statistic: AA vs TT: 7.67 (0.0056)** Genotype: TT; No. of patients: 84 No. of events:2 Survival Time, mo (95% CI): 30 (29–30) Log-Rankk Statistic: AT vs TT: 3.54 (0.0600) Cox Proportional Hazards Regression Model TT: n = 84; p = n.a.; OR (95% CI) = 1 AT: n = 132; p = 0.066; OR (95% CI) = 4.09 (0.91–18.32) **AA: n = 60; p = 0.023; OR (95% CI) = 5.93 (1.28–27.48)**
Umeda et al, (2008) [17]	Intervention pre/post study	n = 44: n = 22: Arg/Arg (21 Male, 95%, 73±8 yrs) Smoking status: Ex/current: 21/1 Pack-years: n.a FEV_1_: (%pred) Median 50.7 Interquartile range (38.1–67.2) COPD grades(I/II/III/IV): n.a n = 22: non-Arg/Arg (19 Male, 86%, 68±8 yrs) Smoking status: Ex/current: 21/1 Pack-years: n.a FEV_1_ (%pred) Median 63.9 Interquartile range (42.3–89.0) COPD grades(I/II/III/IV): n.a	Japaanese	Quality of life	SGRQ	ADRB2	rs1042713	**Total Score:** **Arg/Arg: -16.9; non-Arg/Arg: -8.1** **P = 0.05** **Impact score:** **Arg/Arg: -19.8; non- Arg/Arg: 22** **p<0.001**
Yang et al, (2003) [20]	Observational study	n = 82 (52 Male,63%,69±8 yrs) Smoking status: Ex/current: n.a/n. a Pack-years: Median 54 IQR (39–66) FEV_1_:(%pred): 41±14 COPD grades(I/II/III/IV): n.a	Australian	Exacerbation frequency	Telephone call	MBL2	rs1800450	**OR 4.9; 95% IC: 1.7–14.4; p = 0.0037,** **p**_**corrected**_ **= 0.011**
Zhang et al, (2015) [26]	Intervention pre/post study	n = 219 (194 Male, 88.6%,70.2±7.2 yrs) Smoking status: n.a. Pack-years: 28.1±7.2 FEV_1_:(%pred): 51.8±15.9 COPD grades(I/II/III/IV): n.a	Chinese	Respiratory Health status	SGRQ	EPHX1	rs1051740 (slow allele) rs2234922 (fast allele)	Total score Slow activity group: Baseline: 36.9±12.1; After NAC: 35.1±12.7 p>0.05 Fast activity group: Baseline: 38.0±13.1; After NAC: 35.8±14.8 p>0.05 **Symptom score** **Slow activity group:** **Baseline: 40.9±12.3; After NAC: 37.8±13.1** **p = 0.033** Fast activity group: Baseline: 41.7±13.5; After NAC: 38.7±15.4 P>0.05 Activity score Slow activity group: Baseline: 47.2±12.1; After NAC: 45.8±12.8 p>0.05 Fast activity group: Baseline: 48.7±13.3; After NAC: 49.1±14.0 p>0.05 Impact score Slow activity group: Baseline: 26.3±10.9; After NAC: 24.9±11.5 p>0.05 Fast activity group: Baseline: 28.1±11.7; After NAC: 26.5±12.1 p>0.05
Zhang et al, (2015) [18]	Intervention pre/post study	n = 368 L+: n = 154 (140 Male, 90.9%, 68.7±6.3 yrs) Smoking status: Ex/current: 123/31 Pack-years: 24.5±6.6 FEV_1_: (%pred): 56.4±14.8 COPD grades(I/II/III/IV): n.a L-: n = 214 (193 Male,90.2%,69.3±5 yrs) Smoking status: Ex/current: 170/44 Pack-years: 24.7±5.6 FEV_1_: (%pred) 56.6±19.2 COPD grades(I/II/III/IV): n.a	Chinese	Respiratory health status	SGRQ	HO-1	(GT)n polymorphism:	Total score Baseline—L+ group:35.0±11.6; L- group: 35.1±10.7 16 weeks—L+ group:35.7±9.6; L- group: 35.9±11.3 32 weeks—L+ group:36.2±11.2; L- group:36.4±12.4 48 weeks—L+ group:37.4±10.4; L- group: 37.8±9.0 p = 0.18 **Activity score** **Baseline—L+ group:46.7±13.9; L- group:46.2±14.5** **16 weeks—L+ group:46.5±14.1; L- group: 46.3±11.0** **32 weeks—L+ group:46.8±8.3; L- group: 46.7±12.2** **48 weeks—L+ group:46.3±11.5; L- group: 47.4±15.5** **p = 0.02** Symptom score Baseline—L+ group:40.5±10.8; L- group: 40.1±12.3 16 weeks—L+ group:40.8±8.4; L- group: 40.8±15.1 32 weeks—L+ group:41.1±6.9; L- group: 41.6±10.3 48 weeks—L+ group:41.7±13.7; L- group: 41.6±9.7 p = 0.86 Impact score Baseline—L+ group:25.6±10.1; L- group: 26.0±11.4 16 weeks—L+ group:25.9±7.1; L- group: 26.5±8.6 32 weeks—L+ group:25.7±11.3; L- group: 26.9±10.5 48 weeks—L+ group:25.9±8.9; L- group: 26.7±13.4 p = 0.09

All values are presented as mean ± standard error, unless otherwise stated.

M- male; yrs- years; SNP- single nucleotide polymorphism; FEV_1_- forced expiratory volume in 1 second; l- liters; %pred- % predicted; COPD- chronic obstructive pulmonary disease n.a- not applicable; SE- standard error; IRR- Incidence rate ratio; IC- Confidence interval; OR- Odds ratio; SGRQ- St. George Respiratory Questionnaire; BCSS- Breathlessness, cough and sputum scale; HADS—Hospital anxiety and depression scale. ´

Significant results presented in bold.

The most frequent genetic variants were the mannose-binding lectine (*MBL2*) gene variants (n = 3) [14, 20, 22] and the beta-2 adrenoceptor gene (*ADRB2*) polymorphisms (n = 2) [16, 17]. Other genetic variants observed were group component (*GC*) single nucleotide polymorphisms (SNPs) (n = 1) [25], *HHIP/CHRNA/FAM13A* variants (n = 1) [15], *SERPINA* 11478 G>A variant (n = 1) [23], 25-hydroxyvitamin D receptor (*VDR*) polymorphisms (n = 1) [24], C-C motif chemokine ligand 1 (CCL1) SNPs (n = 1) [19], serotonin transporter gene variant (*SLC6A4*) (n = 1) [21], heme-oxygenase (*HO-1*) gene promotor polymorphism (n = 1) [18] and epoxide hydrolase 1 (*EPHX1*) polymorphisms (n = 1) [26].

The PRO most assessed was the exacerbation frequency (n = 7) [14, 15, 20, 22–25], followed by health-related quality of life (n = 4) [16–18, 26], anxiety and depression (n = 1) [21], exacerbation severity [19] and breathlessness, cough and sputum (n = 1)[16]. Exacerbation frequency was assessed using daily diaries (n = 3)[23–25], phone calls (n = 3)[14, 15, 20], questionnaires (n = 1)[15] and patient interviews (n = 1)[22]. Anxiety and depression were assessed using the Hospital Anxiety and Depression Scale (HADS)(n = 1)[21], and health-related quality of life and respiratory health status using the St George Respiratory Questionnaire (SGRQ) (n = 4) [16–18, 26]. Exacerbation severity was also assessed with questionnaires (n = 1)[19] and finally breathlessness, cough and sputum were assessed using the Breath Cough and Sputum scale (BCSS) (n = 1)[16].

### Synthesis of results

#### Genetic variants and exacerbation frequency

Two coding GC SNPs rs4588 and rs7041 [25], 5 SNP’s and 7 haplotypes from *MBL2* gene have been investigated for associations with exacerbation frequency [14, 20, 22]. Only patients with C allele at the rs4588 polymorphism (C/C: 83 patients; A/C: 45 patients; A/A: 7 patients (p = 0.0048)) [25], 3 *MBL2* SNP’s and 1 haplotype were found significantly more prevalent in frequent exacerbators (p<0.01) [14, 20, 22]. *HHIP*, *FAM13A and CHRNA3/5* SNPs were also assessed, however, only the rs13118928 SNP of the *HHIP* gene was found to be associated to previous and prospective exacerbations (Incidence Rate Ratio = 0.877; p = 0.015 and IRR = 0.906;p = 0.024, respectively) [15]. *SERPINA1* 11478 G>A variant [23] and 25-hydroxyvitamin D receptor (*VDR*) polymorphisms [24] were not associated with exacerbations frequency (α_1_-antitrypsine: p = 0.75; VDR polymorphisms: rs1544410: p = 0.43; rs731236: p = 0.64 rs2228570: p = 0.87) [23, 24]

#### Genetic variants and exacerbation severity

The A allele from the rs2282691 SNP in *CCL1* gene was found to be a risk allele for severity of exacerbation (OR 5.93; p = 0.023) [19].

#### Genetic variants and depression

Only the rs3794808 SNP from the 5 *SLC6A4* gene polymorphisms (rs3794808; rs140701; rs140700; rs2020939; rs2020936) was considered significantly associated with HADS depression score in patients with COPD (p = 0.022)[21].

#### Genetic variants and health-related quality of life

The impact of *HO-1* and *EPHX1* polymorphisms on treatments with N-acetylcysteine (NAC) was assessed [18, 26]. Better health-related quality of life, assessed with the SGRQ, was found in patients without the L allele (L-) of HO-1 gene, which is a (Gt)_n_ polymorphism, than in those with the L allele (L+) relative to the activity score of SGRQ (SGRQ activity score: Baseline: 46.2±14,5; 16 weeks: 46.3±11.0; 32 weeks: 46.7±12.2; 48 weeks: 47.4±15.5; p = 0.02)[18]. Additionally, patients having the slow activity group of the EPHX1 genotype (based on exon 3 polymorphism) also revealed better health-related quality of life than those having the fast activity group for the symptom score of SGRQ (Slow activity group: baseline: 40.9±12.3; after NAC: 37.8±13.1; Fast activity group: baseline: 41.7±13.5; after NAC 38.7±15.4; p<0.05) [26].

The impact of the Gly16Arg polymorphism of the ADRB2 gene on health-related quality of life HRQOL was also assessed. Significant differences were observed in all domains and total scores of SGRQ between both genetic groups (Arg/Arg and non- Arg/Arg), however only the impact and total scores were significantly different in patients with the Arg/Arg genotype (total score: -16.9 vs. -8.1, p = 0.005; impact score: -19.8 vs. -2.2 p<0.001)[17]. No significant associations were found when investigating impact of the ADRB2 polymorphism on treatment effect with budesonide/formoterol I(p = 0.909) and II(p = 0.648) on SGRQ[16].

#### Genetic variants and breathlessness, cough and sputum

No significant association was found between the Gly16Arg possible genotypes of ADRB2 and the scores for the BCSS scale (p>0.05)[16].

## Discussion

This was the first systematic review to explore associations between genetics and PROs in patients with COPD. The 13 studies included reported on 12 genetic variations positively associated with 5 distinct PROs, i.e., exacerbation frequency and severity, depression, health related quality of life and symptoms (breathlessness, cough and sputum).

Most studies (n = 7/13) assessed the association of specific genetic variants with exacerbation frequency. This is an important remark since the frequency of exacerbations is strongly associated with patients’ functional and physiological deterioration [27], reduced health related quality of life [28] and substantial morbidity and mortality [29]. *MBL2* was the gene mostly associated with frequency of exacerbations (3/13) [14, 20, 22]. Several polymorphisms of *MBL2* gene play important roles in the innate immunity as it encodes for mannose-binding lectine. The mannose-binding lectine is a pattern-recognition receptor that binds to the sugar structure presented in various microorganisms[30]. Specific polymorphisms of the *MBL2* gene have been found responsible for causing a decreased production of MBL (MBL-deficient genotype), and this has been associated with an increased risk of exacerbations. In fact, high MBL levels presented in serum have been associated with increased survival in COPD [14]. Thus, *MBL2* polymorphisms seem to be promising biomarkers to detect those with more susceptibility to exacerbations and good candidates for assessment with PRO-based approaches.

Polymorphisms in the *GC* and *VDR* genes causing deficits of vitamin D (associated with several comorbidities, such as osteoporosis or skeletal muscle dysfunction, in patients with COPD[31]), have also been connected to the frequency of exacerbations. Nevertheless, a careful interpretation of the literature is needed since both significant and non-significant associations between GC polymorphisms rs4588 and rs7041 or *VDR* (Bsm, Taql, Fokl) polymorphisms with frequency of exacerbations and lack of vitamin D have been reported [25, 32, 33]. Additionally, many non-genetic factors may also lead to vitamin D deficiency, namely the absence of sun exposure, vitamin D retention on body fat or other social/cultural factors[31]. Therefore, future studies are yet needed to enhance our understanding of the relationship between these polymorphisms, vitamin D deficiency and PRO in COPD.

Exacerbation severity has been significantly associated with a *CCL1* allele for rs2282691 in one study [19]. However, the authors’ definition of exacerbation severity can be arguable, as they used death as endpoint. It is known that the severity of an exacerbation is not defined by mortality but rather by symptoms and number and length of hospitalizations in the most severe cases[34]. Since the authors have recorded patients’ main symptoms, it would be interesting to assess if the reported A allele for rs2282691 was also associated with those and if the T allele actually conferred protection to acute exacerbations of COPD, as suggested. Other option would be to explore the correlations between rs2282691 variant with specific instruments, such as the Exacerbations of Chronic Pulmonary Disease Tool (EXACT-PRO)[35] to assess the severity of exacerbations. This analysis would be essential to identify patients with higher predisposition for more severe exacerbations which would allow them to be targeted for more timely and directed monitoring and intervention.

Depression was found to be associated with the rs3794808 variant, affecting the *SLC6A4* gene[21]. It is known that depression presents a strong linkage to nicotine dependence[36] and *SLC6A4* is strongly associated with the pathophysiology of tobacco use, namely at the level of serotonin reuptake. Therefore, it would be expected that specific genetic variants of this gene would play an important role on nicotine dependence and consequently depression in ex/current smokers[37]. However, different SNPS of the *SLC6A4* gene [38] and other genes such as *THSD4*, *CHRNA*, *CYP2A6*[3] have also been associated with depression in COPD. Thus, it would be valuable to confirm those associations populations patients with COPD with different characteristics, such as smokers and non-smokers, to decide if future therapies should take these genes into consideration. Currently, the most effective therapy to combat anxiety and depression in patients with COPD is pulmonary rehabilitation (PR), which has been shown to be significantly effective by reducing the levels of depression and anxiety symptoms in patients with this disease [39]. However, PR is an expensive therapy and access to it is highly limited [40]. Therefore, genetics may be used to signal priority patients to PR, and thus optimize human and financial resources in managing COPD.

Four studies investigated *ADRB2*[16, 17], *Ho-1*[18] and *EPHX1*[26] association with health related quality of life. The Gly16Arg polymorphism of *ADRB2* has been indicated as a risk factor for COPD[41]. However, different results regarding its association with health-related quality of life emerged from our systematic review. Umeda et al., showed that patients with COPD and the Arg/Arg genotype presented better health related quality of life in treatments with tiotropium[17] whereas no significant associations was found in the study of Bleecker et al. for the same polymorphism and budesonide/formoterol treatment [16]. The most obvious explanation is the substance used in the treatments, since other studies using other LABAs (long-acting b2-agonists) and LAMAs (long-acting muscarinic antagonists) have also presented no associations [42]. As for *Ho-1* and *EPHX1* polymorphisms, both genes presented significant associations with SGRQ activity and symptoms sub-scores in patients with COPD that were treated with N-acetylcysteine (NAC)[18, 26]. Pharmacogenetics studies are of significant importance, since they investigate how genes affect a patient’s response to drugs. This knowledge facilitates health-care by identifying patients that will respond differently to treatments. Future studies assessing health related quality of life may also include these genetic variants reported as being protective against COPD[43], since they may play an important role on patients’ quality of life.

A final important aspect to emphasize is the ethnicity of the study populations. In this study we intended to summarize the genetic variants associated with PROS in patients with COPD. However, we observed that this systematic review included nine different ethnic groups from which the majority (n = 7/13) were conducted in Asian countries. This may explain the difficulty in obtaining similar results among different populations. For this reason, the ethnicity was also indicated in Table 2.

PROs are increasingly being understood as excellent instruments to translate a range of outcomes that spirometry cannot express, such as symptoms and patients’ perspective of treatment[44]. However, there is a massive number of PROMs (patient-reported outcomes measures) that were not found in this systematic review, and yet allow to assess other fundamental PROs such as mood, social and sexual life [6]. Also, it was shown that genetics play a key role not only in the predisposition to the disease but also in common COPD-related comorbidities. Thus, further studies should be conducted to re-enforce the present knowledge and assess the genetic influence on other dimensions of patients’ lives.

### Limitations

This review has some limitations that need to be acknowledged. Firstly, the definition of PROs (although published) has not been used as a primary outcome in some of the included studies. Thus, few studies gave emphasis to it, giving priority to clinical outcomes which may have led to significant loss of studies and information. However, to minimize this problem we performed a meticulous choice of keywords to diminish the number of missed studies. Secondly, the constant changes in the definition of exacerbation in the GOLD[1] may also result in loss of studies over the years. We overcame this difficulty by enclosing studies which included participants that had reported exacerbations independently of the definition used at the time. Thirdly, studies used different methodologies to assess similar or different PROs and consequently, prevented the realization of meta-analysis.

## Conclusions

This was the first systematic review to explore associations between genetics and PROs in COPD. Although a limited number of PROs have been successfully related to genetic variations, findings suggest that a significant association between specific genetic variants and the frequency and severity of exacerbations, health-related quality of life and depressive symptoms may exist. Thus, further research is needed to confirm these results and to assess the possibility of association of other genetic variants with other PROs in patients with COPD, since this may enhance our understanding and management of this disease.

## Supporting information

S1 Table Search strategy. (PDF)

S2 Table PRISMA checklist. (PDF)
