# Supplementary material for: Genetic profile and patient-reported outcomes in chronic obstructive pulmonary disease: A systematic review
Source: PLoS One. 2018 Jun 21;13(6):e0198920. doi: 10.1371/journal.pone.0198920 (PMC6013101; doi:10.1371/journal.pone.0198920)
Supplement: S1 Table — (PDF) [file pone.0198920.s001.pdf]

Pubmed
[(COPD OR "chronic obstructive pulmonary disease" OR emphysema OR "chronic bronchitis") AND ("genetic associations" OR "genetic profile" OR "genetic analysis" OR gene) AND (dyspnea OR dyspnoea OR breathlessness OR fatigue OR cough OR depression OR anxiety OR "daily living" OR "quality of life" OR mood OR "well-being" OR “frequency of exacerbation” OR exacerbations OR "hospital admissions" OR "hospital length of stay" OR "acute exacerbations" OR “physical activity” OR "physical fitness" OR “physical function” OR “sputum production” OR phlegm OR pain OR “patient-reported outcomes” OR “patient-centered outcomes” OR “patient-centered outcomes”)] [Limit to: 1950-2016].
Scopus
[(COPD OR "chronic obstructive pulmonary disease" OR emphysema OR "chronic bronchitis") AND ("genetic associations" OR "genetic profile" OR "genetic analysis" OR gene) AND (dyspnea OR dyspnoea OR breathlessness OR fatigue OR cough OR depression OR anxiety OR "daily living" OR "quality of life" OR mood OR "well-being" OR “frequency of exacerbation” OR exacerbations OR "hospital admissions" OR "hospital length of stay" OR "acute exacerbations" OR “physical activity” OR "physical fitness" OR “physical function” OR “sputum production” OR phlegm OR pain OR “patient-reported outcomes” OR “patient-centered outcomes” OR “patient-centered outcomes”)] [Limit to: 1960-2016].
Web of Science
[(COPD OR "chronic obstructive pulmonary disease" OR emphysema OR "chronic bronchitis") AND ("genetic associations" OR "genetic profile" OR "genetic analysis" OR gene) AND (dyspnea OR dyspnoea OR breathlessness OR fatigue OR cough OR depression OR anxiety OR "daily living" OR "quality of life" OR mood OR "well-

being" OR “frequency of exacerbation” OR exacerbations OR "hospital admissions" OR "hospital length of stay" OR "acute exacerbations" OR “physical activity” OR "physical fitness" OR “physical function” OR “sputum production” OR phlegm OR pain OR “patient-reported outcomes” OR “patient-centered outcomes” OR “patient-centered outcomes”)] [Limit to: 1900-2016].
